# Exponential growth of private coastal infrastructure influenced by geography and race in South Carolina, USA

**DOI:** 10.1038/s41598-024-59740-x

**Published:** 2024-04-20

**Authors:** Jeffrey Beauvais, Scott N. Markley, James E. Byers

**Affiliations:** 1 Odum School of Ecology, University of Georgia, 140 E. Green St., Athens, GA 30602 USA; 2 Department of City and Regional Planning, Cornell University, 216 West Sibley Hall, Ithaca, NY 14583 USA

**Keywords:** Environmental social sciences, Environmental impact, Urban ecology

## Abstract

Homeowners in coastal environments often augment their access to estuarine ecosystems by building private docks on their personal property. Despite the commonality of docks, particularly in the Southeastern United States, few works have investigated their historical development, their distribution across the landscape, or the environmental justice dimensions of this distribution. In this study, we used historic aerial photography to track the abundance and size of docks across six South Carolina counties from the 1950s to 2016. Across our roughly 60-year study period, dock abundance grew by two orders of magnitude, mean length of newly constructed docks doubled, and the cumulative length of docks ballooned from 34 to 560 km. Additionally, we drew on census data interpolated into consistent 2010 tract boundaries to analyze the racial and economic distribution of docks in 1994, 1999, 2011, and 2016. Racial composition, measured as the percentage of a tract’s population that was White, positively correlated with dock abundance in each year. Median household income and dock abundance were only correlated in 2011. Taken together, these metrics indicate the growing desire for direct estuary access, however, that access does not appear to be equally spread across racial groups. Because docks enhance estuarine access and demarcate private property, our study provides longitudinal insights into environmental justice concerns related to disparate private property ownership. We found a persistent correlation between the racial characteristics of an area and dock abundance, strongly indicating that White South Carolinians have had disproportionately greater private water access for the past two decades.

## Introduction

Coastal environments are dynamic, ecologically rich areas that provide numerous ecosystem services for human populations including provisioning of seafood for personal consumption and sale, spaces for recreation, and continuity of cultural practices. To utilize many of these services, individuals must first be able to access them. Access is a complex topic that has received extensive attention in the social sciences, but considerably less work has been done to quantify metrics of access for coastal residents^1,2^. For our purposes, we narrowly define access as the ability to physically enter the marine environment^3,4^. In most US states, navigable waterways (in the case of freshwater rivers) and areas below mean high water (in the case of estuaries) are preserved as public property under the “public trust doctrine”^5^. While these *de jure* (legal) rights of access to water are longstanding and frequently upheld against challenge^6–8^, they also obscure the de facto (actual), lived experience of access. Although estuarine waters generally cannot be privately held, the land bordering them can be privately owned, creating a barrier to access for non-property owners.

In many areas, “water access infrastructure” (WAI) such as docks, piers, boat landings, and marinas determine where individuals can access coastal ecosystems and what activities they can engage in at those sites. Private docks augment individual access to estuaries by providing entry through their fixed structure (particularly in soft-sediment ecosystems) and a place to store and launch vessels. More importantly, because most docks in South Carolina are built on personal property^9^ they can act as a conservative proxy for the number of waterfront private property parcels in an area. This provides researchers with a useful, easily identifiable feature to quantify the minimum extent of shoreline privatization. Thus, analyzing how private docks (and therefore private waterfront property) are distributed across a landscape can help address the potential disconnect between legal and functional access to coastal waters, as well as indicate land uses and ownership bordering the coastline.

By centering the distinction between *de jure* and de facto experiences of water access, we shift our focus towards questions of who enjoys these benefits through private ownership of waterfront land. The field of environmental justice (EJ) has long noted that environmental benefits and burdens are frequently distributed along lines of social hierarchy, such as those entrenched in race and class. EJ scholars and activists have compellingly shown that a variety of environmental burdens including toxic substance storage sites^10–12^ and poorer air quality^13–15^ are generally concentrated in lower income, non-White communities. Research on beneficial ecosystem services, such as those provided by urban tree canopy, have shown greater cover in more affluent, White neighborhoods^16,17^; however, local context is important in driving the strength and direction of those effects^18^. Comparatively few works have directly quantified access in coastal systems through an EJ framework, but studies have found mixed results with respect to the influence of race and income on public access points^1,2,19^. To our knowledge, only one other study has focused on the socioeconomic distribution of private water access in the Southeastern US^9^.

Apart from the spatiality of environmental benefits and burdens, temporal changes are another critical aspect of EJ research. In the early years of EJ as an academic discipline, skeptical researchers questioned whether documented disparities in exposure to toxic facilities were the result of low-income and non-White communities moving into these areas after the sites were established, rather than disproportionate siting of toxic facilities in non-White communities^20^. As the field developed, EJ research has generally demonstrated that facilities were sited in majority non-White and low-income communities at the time of their founding and that the establishment of toxic facilities caused negligible changes to the racial composition of surrounding communities through time^21–25^. Moreover, other researchers have argued that focusing solely on individual siting decisions improperly conceptualizes environmental injustices as a product of deliberate, malicious acts, instead of a consequence of structural racism in housing, industrial, land use, and urban development policy that drives where different populations live and work^11,23,26^. Longitudinal studies, or those that track the same suite of variables in a consistent area over time, can provide a powerful spatiotemporal analysis of the progression of environmental injustices^24^ and help reveal processes embedded in broader systems of social stratification that produce environmental injustices in the first place^26^. Thus, while notably more difficult to conduct, longitudinal EJ research provides additional context for how different communities experience environmental injustices and how injustices progress through time.

This study builds on previous research we conducted on 2016 data of public and private WAI distribution in coastal South Carolina^9^. We identified nearly 12,000 private docks across six counties in that study, which inspired us to examine the historical development of these structures. While some studies have examined the current extent of coastal infrastructure in marine systems at the local^27,28^, national^29–31^, and international^32,33^ scale, few focus on the historical progression of coastal infrastructure^34^. Additionally, most investigations have focused on large industrial infrastructure or armoring and do not quantify the extent of private, personal WAI. Despite not being put to heavy industrial use, personal docks have been shown to have numerous ecological effects, including shifting community composition^35,36^, enhancing the spread of introduced species^37^, and shading macrophytes^38,39^. Given their pervasiveness, density, and potential ecological effects, we believe understanding the temporal dynamics of docks provides information on key historical inflection points in coastal development, which can help guide both research and management of human infrastructure in these ecosystems.

Apart from their ecological dimensions, our prior work explicitly focused on environmental justice implications of public and private WAI. We found that racial composition strongly influenced dock abundance in census block groups while household income was not correlated. As noted, however, this research only considered a single year of data (2016) and the historical development of private water access in South Carolina remained unclear. Given our joint interest in both the ecological and EJ impacts of private docks, we address two primary questions in this study. First, how has dock abundance and length changed along the South Carolina coast from the 1950s to 2016? We predict that dock abundance and lengths will increase through time, with abundances demonstrating punctuated growth spurts (“start and stop”) that follow larger trends in the broader economy. Second, has the distribution of docks with respect to the economic and racial composition of census tracts changed from 1994 to 2016? We expect a positive relationship between both the percentage of a census tract’s White population and median household income with dock abundance. To our knowledge, this study represents the first longitudinal analysis of private WAI.

## Methods

### Study system and extent

**Figure 1:**
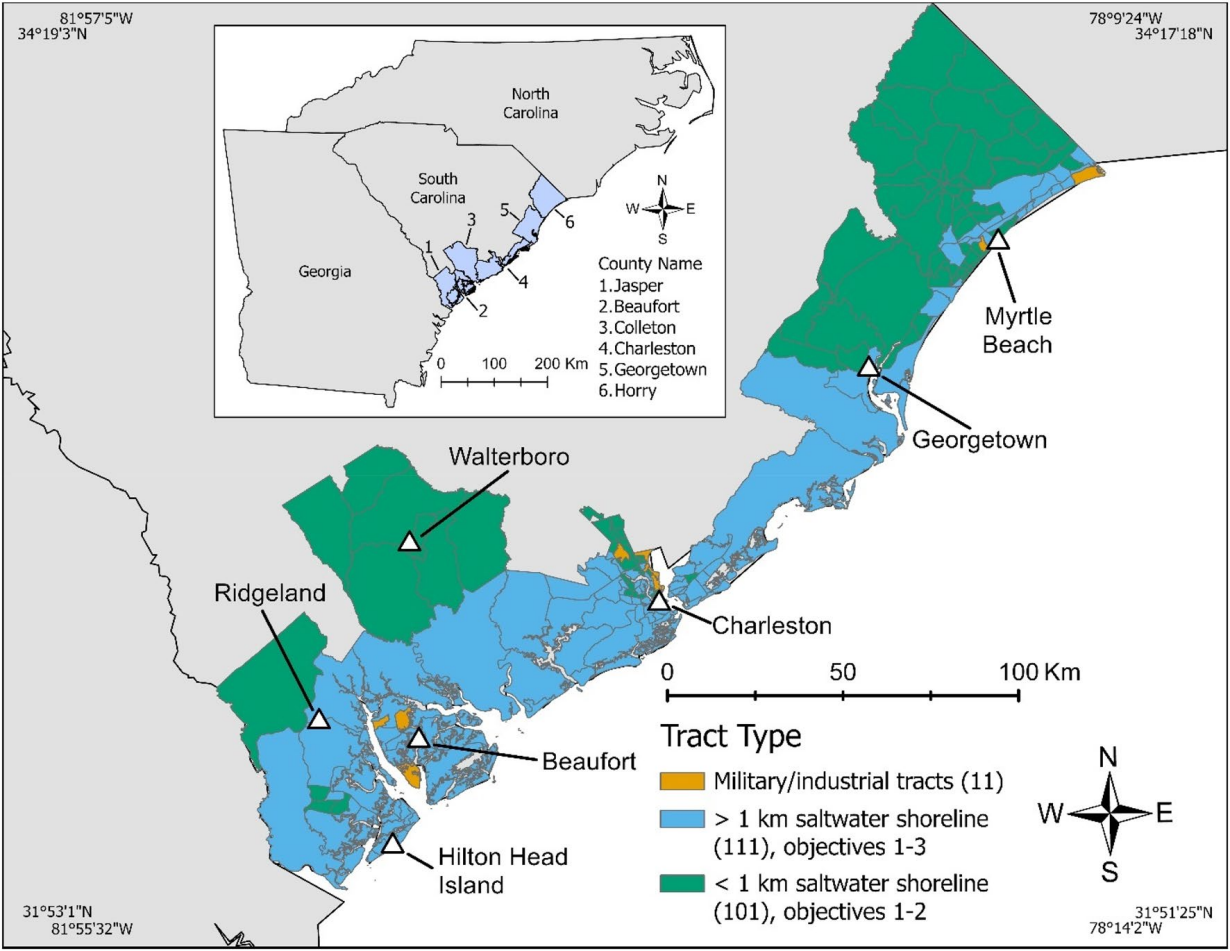
The main map shows census tracts in the study area. Orange tracts are primarily military and industrial installations and we did not count docks in these areas. We only included blue tracts in objective 3, but blue and green tracts are used in all objectives. The numbers in parentheses in the legend represent the number of tracts in each category. The inset map (top left) shows South Carolina and neighboring states. Major cities in South Carolina are marked by white triangles and associated text. Main map scale 1:1,250,000, county inset map scale 1:8,250,000. The figure was created in ArcGIS Pro Version 2.9^42^. County and tract boundaries were downloaded from the National Historical GIS^45^.

We conducted our research in the six counties along the South Carolina coast (Fig. 1, inset map). The waterways of coastal South Carolina are composed of estuaries that form the transition to interior brackish and freshwater rivers, typical of other Southeastern US states. In South Carolina, all areas below mean high water are public property, however, as discussed in the introduction, this legal status does not ensure functional access to coastal waters. Our study builds on prior research we conducted on dock distribution in this same area and further system description can be found therein^9^.

We collected and analyzed dock data at the census tract level. Census tracts are a sub-county geographic unit used by the Census Bureau to summarize census data. The protocol used by the Census Bureau to designate tracts has changed over time but stabilized following the establishment of tracts across the entire United States for the 1990 census^40,41^. Since the 1990s, tracts have been designed to encompass 1200–8000 people and generally follow visually identifiable, permanent features of the landscape like roads, bodies of water, and above-ground utility lines^40^. Our six counties initially consisted of 223 tracts (Fig. 1, main map). Since we are primarily interested in private docks in residential areas, we did not record docks in 11 tracts, bringing our total study area down to 212 tracts. Of these 11 tracts, four were composed entirely of military bases/housing, one made up the Myrtle Beach airport, and five made up the Port of Charleston (Fig. 1, orange tracts). We also removed one tract in Horry County that was an extremely high outlier in dock abundance and demonstrated a unique development pattern not replicated anywhere else in South Carolina.

### Objectives

We assessed our research questions through three objectives: (1) enumerate new, removed, and existing docks in the 212 tracts across our study area (green and blue tracts, Fig. 1) across all years (1950s-2016); (2) analyze how the length of new docks has changed over time in the 212 tracts across our study area (1960s-2011); and (3) analyze how existing dock abundance is distributed with respect to the racial and economic composition across the 111 tracts with more than 1 km of saltwater shoreline (1994–2016). The date ranges and geographic extent of our objectives differ because we were constrained by years where necessary data was available. We define “new” as docks we could verify were constructed between two years of imagery, “removed” as docks that went missing between two years of imagery, and “existing” as the number of extant docks in each year of imagery (calculated as existing docks from the prior year of imagery + new docks—removed docks).

### Data collection—response variables

We gathered data on docks from three sources of historical aerial photographs of the South Carolina coast (Table 1). We obtained imagery for years prior to 1990 from the University of South Carolina Thomas Cooper Library (“pre-1990 imagery”). Pre-1990 image quality varied from 1.25 to 4.5 m resolution, with greater resolutions typically found in later years. The South Carolina Department of Natural Resources (DNR) provided statewide, 1-m resolution color infrared imagery from 1994 to 1999. We downloaded 1-m resolution 2011 imagery from the US Department of Agriculture National Agriculture Imagery Program’s Geospatial Data Gateway (https://nrcs.app.box.com/v/gateway/folder/19350726983). We present the years of imagery for each county in Table 1. Additionally, we also used 2016 data from our prior study of South Carolina docks^9^. We did not gather the 2016 data from imagery, instead we acquired it as pre-digitized dock polygons from the Ocean & Coastal Resource Management division of the South Carolina Department of Health and Environmental Control (https://sc-department-of-health-and-environmental-control-gis-sc-dhec.hub.arcgis.com/).

**Table 1:** Table of imagery years, corresponding census decades, and sources.

County	Imagery Year	Census Decade	Source	County	Imagery Year	Census Decade	Source	County	Imagery Year	Census Decade	Source
Beaufort	1955	**1960**	USC	Charleston	1949	1950	USC	Colleton	1948	1950	USC
	1959	**1960**	USC		1963	1960	USC		1957	**1960**	USC
	1972	1970	USC		1973	1970	USC		1963	**1960**	USC
	1979	1980	USC		1979	1980	USC		1973	1970	USC
Georgetown	1957	**1960**	USC	Horry	1963	1960	USC	Jasper	1955	1960	USC
	1963	**1960**	USC		1973	1970	USC		1965	**1970**	USC
	1973	1970	USC		1983	1980	USC		1970	**1970**	USC
	1980	1980	USC						1979	1980	USC
All counties	1994	1990	DNR								
	1999	2000	DNR								
	2011	2010	NAIP								
	2016	2019	OCRM								

Bold text represents two imagery years that correspond to the same census decade. Note that we downloaded the 2016 data as pre-made digitized polygons of docks, not aerial imagery.

*USC* University of South Carolina Thomas Cooper Library, *DNR* South Carolina Department of Natural Resources, *NAIP* United States Department of Agriculture National Agriculture Imagery program, *OCRM* South Carolina Ocean and Coastal Resource Management division.

For all imagery, we systematically searched for docks in every county/year combination in ArcGIS Pro Version 2.9^42^ by sequentially examining individual photographs for the pre-1990 imagery or individual tracts for the composite 1994, 1999, and 2011 imagery. While checking imagery, we kept a polygon layer showing the outline of waterbodies open to ensure that we checked all locations where a dock could be built. Because we treat docks as a conservative proxy for private, waterfront property, we took steps to identify and omit public docks (i.e., public fishing piers or mooring docks at public boat landings). We provide further details on the protocol taken to omit public infrastructure in the Supplementary Material.

For every dock we encountered we placed one point at the base of the dock (where it connected to the land) and one point at the waterward end of the dock at 1:2,500 scale (Supplementary Fig. S1). We extracted census tract information to the base point of every dock to obtain tract-level dock counts. Because we worked with a longitudinal data set and most docks are preserved from year to year, we opted to analyze each county in chronological order and build upon the dock data from the prior year of imagery (e.g., the completed dock point layer for Beaufort 1955 served as the starting point for 1959, 1959 served as the starting point for 1972, etc.). This approach greatly reduced the labor of identifying and labeling the same docks, aided in identification of new and removed docks, and allowed us to determine the longevity of dock occupation at a given location (“dock presence”).

Because of our continuous approach to counting docks, we had to determine whether points from prior years of imagery mapped to docks in sequential imagery years. Small variations in georeferencing historic imagery caused some points placed on docks to shift slightly across years (Supplementary Fig. S1, S2). When this occurred, we referenced imagery from prior years and relied on the overall number of docks in the scene, dock shape, angle of points, placement relative to consistent objects across images, and maximum displacement of points between imagery years to determine which docks were maintained, new, and removed. We describe full details on these assignment rules, as well as quality control and assessment protocols to ensure consistency across researchers, in the Supplementary Material. Because the 2016 data came as pre-drawn polygons instead of imagery, it proved enormously difficult to match this with our point data set and we only report the existing number of docks for 2016.

We calculated dock lengths using the “geosphere” package^43^ in R Version 4.2.1^44^. We noted 167 times where a dock in the same location underwent obvious expansions, replacement, or experienced damage that altered the length of the dock by more than 10 m. This is an underestimate of these occurrences, because we did not quantify these situations before 1994. If a dock did change in length, we marked the dock from the prior decade as removed and treated the modified dock as new to account for this length change. In our analysis of counts, however, we ignored these instances, because these changes affected the same structure on the same private lot. Our length data does not include the 2016 data because the polygon format prevented us from calculating dock lengths.

### Data collection—census and landscape variables

We selected our predictor variables for the socioeconomic analysis based on our system-level knowledge and commonly included variables in EJ studies. We obtained our demographic variables from different data sources depending on the research objective. For objective 1, we obtained county-level data on total population and number of housing units for each decennial census from 1950 to 2020 from the National Historic GIS (NHGIS)^45^. We then aggregated this data across all counties in each decade to obtain population and housing unit counts across the entire study area. We used county-level census data because most counties in our study area did not contain census tracts until 1990.

For objective 3, we obtained historical census tract variables from the NHGIS and Historical Housing Unit and Urbanization Database (HHUUD10)^46^. These variables include the housing unit and population counts, median household income (MHI), percentage White population, and homeownership rate for the 1990, 2000, and 2010 decennial censuses and the 2019 five-year ACS sample. We used the 2019 ACS five-year sample because tract-level data from the 2020 decennial census was not available at the time of analysis. Additionally, we only analyzed data from 1990, 2000, 2010, and 2019 for objective 3 because 1990 was the first census in which all our study counties contained tracts. Because census tracts are redrawn every ten years, these variables need to be harmonized into a single consistent tract geometry to allow for historical comparison. Data from the 2010 decennial census and 2019 five-year ACS sample are already in the same census tract vintage, so no data interpolation is necessary for those years. However, the 1990 and 2000 data are not compatible. Fortunately, the makers of HHUUD10 and the NHGIS time series dataset have already conducted the requisite areal interpolation. Specifically, HHUUD10 provides ready-made housing unit count data in consistent 2010 census tracts, while the NHGIS provides a series of crosswalk weights to convert the other variables to the same vintage. We thus combine the HHUUD10 housing unit data with NHGIS crosswalk-produced data with the census and ACS data to get our 1990 and 2000 tract variables into the 2010 tract vintage.

In addition to our demographic variables, we also collected data on shore length because our prior work showed it was strongly, positively correlated with dock abundance^9^. We relied on shoreline estimates derived from this previous study to calculate the length of beach, freshwater, and deep-water saltwater/brackish shore length for tracts (excluding isolated ponds and golf course lagoons). These measurements are derived from 2016 30-m resolution land cover rasters from NOAA’s Digital Coast portal (https://coast.noaa.gov/digitalcoast/data/) and South Carolina’s freshwater/saltwater dividing line (https://www.dnr.sc.gov/marine/dividingline.html). We used a single estimate of shore length for all years and therefore assumed that shore length within tracts does not appreciably change over the course of our study. We are comfortable with this assumption given the relatively short time scale (22 years) of our socioeconomic analysis.

### Objective 1—new, removed, and existing dock abundance

We summed the number of new, removed, and existing docks in each county to obtain counts for the entire 212 tract study area. We grouped dock counts into 10-year bins to align with the decadal nature of census data and attempt to ensure each county contained a count in each decade (Table 1). Although this does increase our temporal resolution of dock counts in some bins relative to others, this is a minor issue and we explain our rationale in the Supplementary Material. For removed docks, we noted the decade they were first identified to determine the longevity of dock presence. Like all infrastructure, docks require maintenance, repair, and even occasional replacement. Unless there was a major (> 10 m) change in dock length or orientation, the resolution of our imagery made it impossible to determine when docks underwent routine maintenance or repair. Because of this, we frame dock longevity in terms of the consistent presence of a dock at a given site (i.e., its footprint) and not as a measure of the durability of that specific structure. Our EJ framing focuses on docks as a conservative proxy for private, waterfront property, therefore the presence of a dock at a given location through time is a more relevant measure than whether it is the “same” dock.

Because dock abundance displayed characteristics of exponential growth, we fit a simple exponential curve to existing dock abundance, population, and housing units to compare their rates of growth. Note that because we had to use aggregated county-level data for population and housing units for these calculations we were unable to exclude the 11 military/industrial tracts in which we did not record docks. To visualize where docks are placed on the landscape, we provide a GIF of dock locations (Supplementary Fig. S3, Online Resource 1) and county-level counts for each decade in Supplementary Fig. S4.

### Objective 2—dock lengths

As with the counts, we summarized dock lengths at the level of the entire 212 tract study area, taking the mean length of each dock type (i.e., existing, new, removed) for every decade. Of these three types, we opted to statistically analyze new dock length because only a small proportion of docks are removed each decade (Table 2), thus any changes in existing dock length are likely driven by the construction of new docks. We conducted an ANOVA with post-hoc Tukey tests on new dock lengths and decade to determine whether new docks have gotten longer over time. We provide a visualization of county-level lengths for each decade in Supplementary Fig. S5.

**Table 2:** Table summarizing dock counts across the study area (excluding the 11 military/industrial tracts).

Decade	New docks	Removed docks	Existing docks	% New docks	% Previous decade’s docks removed	Housing units	% Housing unit growth	Population	% Population growth
1950	–	–	197*	–	–	89,568	–	322,668	–
1960	413^†^	44^†^	945	43.70^†^	22.34^†^	118,333	32.12	403,667	25.10
1970	676	148	1,473	45.89	15.66	143,335	21.13	441,785	9.44
1980	736	175	2,034	36.18	11.88	216,033	50.72	532,498	20.53
1990	3232	425	4,841	66.76	20.89	301,621	39.62	621,683	16.75
2000	1691	69	6,463	26.16	1.42	377,964	25.31	742,274	19.40
2010	3780	151	10,092	37.46	2.33	512,871	35.69	905,560	22.00
2019	–	–	11,886	–	–	572,256	11.58	1,077,180	18.95

“New docks” refers to docks we could verify were built in that decade, “removed docks” refers to docks that disappeared in that decade, and “existing docks” refers to all docks during that decade. Because we downloaded the data for the 2019 decade as pre-defined polygons, we could not track which docks were new or removed relative to 2010.

*Only Charleston and Colleton had imagery in the 1950s and Colleton contained no docks. †Because 1960 is the first decade of imagery for Beaufort, Georgetown, Horry, and Jasper, these four columns in 1960 only represent Charleston and Colleton. The “Existing docks” column for 1960 represents all counties. After 1960 all columns report all counties.

### Objective 3—Socioeconomic analysis of dock distribution

Unlike our descriptive analysis of dock counts and lengths across the entire study area, we only included 111 tracts with greater than 1 km of saltwater shoreline in our statistical analysis of dock distribution (Fig. 1, blue tracts). We used this reduced data set because docks cannot be built along the beach and are uncommon on freshwater rivers due to navigability concerns. We chose this 1 km cutoff to ensure that tracts had an appreciable amount of developable shoreline, as we noticed that many tracts with little shoreline were comprised of small embayments that did not contain navigable water. We also removed any tracts with less than 100 housing units from the analysis. We include two tables in the Supplementary Material that quantify how the choice of cutoff affects the number of tracts and docks included in the model (Supplementary Table S1, S2).

As with objective 1, we binned our dock data to align with the decadal census data. Thus, we paired the 1994, 1999, 2011, and 2016 dock data with the 1990, 2000, 2010, and 2019 census data, respectively (Table 1, “All Counties”). For each decade, we fit a separate zero-inflated, negative binomial mixed model with existing dock count as the response variable using the R package “glmmTMB”^47^. We included median household income (MHI) and percentage White as our predictor variables of interest. We corrected MHI for inflation using the “priceR” package^48^, converting all MHI values to 2010 US dollars (USD). We also included the percentage homeownership, population, and shore length in the model as fixed effects. The state of South Carolina requires proof of title for dock permits, so renters are unable to install a dock themselves, and we anticipate that the owners of rental properties are less likely to build and maintain such an expensive amenity. Thus, a higher proportion of rental property in a tract may be correlated with lower numbers of docks. We used total population in place of population density because there was a high correlation between tract land area and shore length, which we already included in the model.

We centered and scaled all fixed effects in the model by their standard deviations to allow for direct comparison of coefficient values. To account for unexplained variance at the tract level and the nesting of tracts within counties, we included both tract and county as random effects. We tested and found no support for an interaction between the variables for income and race in all decades, so we removed the interaction from all models. We did not find any evidence of multicollinearity of predictor variables in any decade (VIF < 3).

We ran similar diagnostics for all models. We examined residuals for homoscedasticity using the “DHARMa” package^49^. We used a Moran’s I test assuming an inverse-distance weighting relationship in ArcGIS Pro to assess whether there was any spatial autocorrelation in model residuals for each decade. For all decades we found no evidence of spatial autocorrelation (1990: z = 1.59, *p* = 0.11; 2000: z = 1.81, *p* = 0.07; 2010: z = 1.29, *p* = 0.20; 2019: z = 1.66, *p* = 0.10). We used likelihood ratio tests to calculate p-values for all fixed effects. To examine the magnitude of fixed effects, we plotted their conditional effects using the “ggeffects” package in R^50^. “ggeffects” generates predicted values of the response variable (dock count) for a focal predictor variable while holding all other fixed effects constant at their mean value. We qualitatively assessed these conditional effects to see if the influence of economic and racial composition changed over time.

## Results

### Objective 1—new, removed, and existing dock abundance

**Figure 2:**
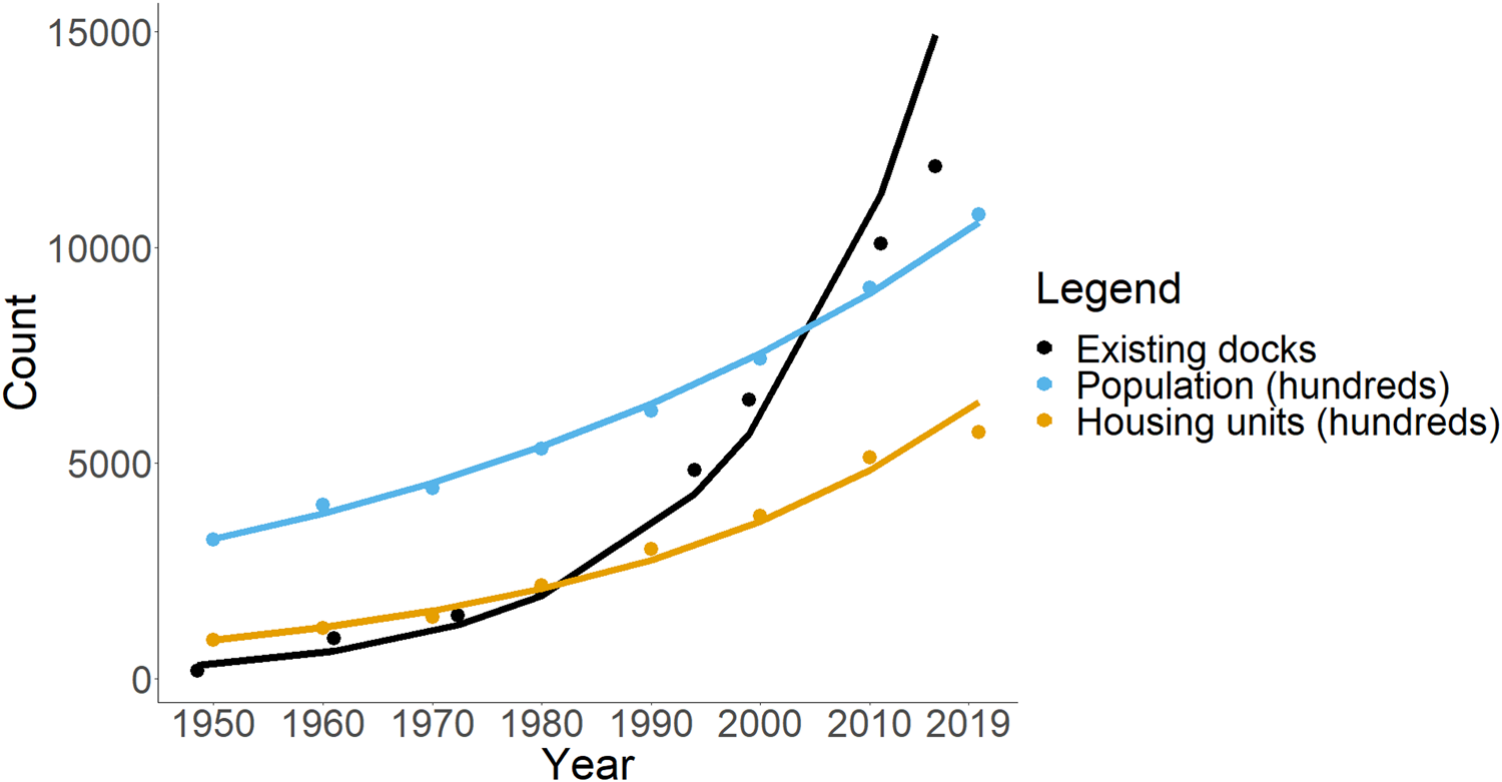
Growth rate of existing docks, human population (in hundreds), and housing units (in hundreds) across all six coastal counties (excluding the 11 military/industrial tracts). Dock counts are fit to mean imagery years (Table 1) for each census decade. Lines represent simple exponential growth curves fit to each variable. Estimated growth rates: r (existing docks) = 0.055, r (population) = 0.017, r (housing units) = 0.028.

Existing dock abundance showed continuous growth throughout the entire study period. Trends in existing dock abundance were largely driven by new dock construction, as a relatively small number of docks were removed each decade (Table 2). The largest relative increase in docks occurred in the 1990 decade (i.e., between 1985 and 1994) where the number of docks in our focal counties increased by a factor of 2.38 (from 2034 to 4841), resulting in two-thirds of all docks in these six counties being new construction (Table 2). The 1990 decade also contained the greatest number (425) and percentage of docks (20.89%) that were removed (when accounting for all counties, Table 2). The number of housing units and total population also steadily increased through time (Table 2). We found that the growth rate of docks (Fig. 2, r = 0.055) was roughly triple and double the growth rates of population (r = 0.017) and housing units (r = 0.028), respectively.

Although we cannot directly measure the durability of a given dock, our analysis demonstrated the longevity of dock presence at a site (Table 3). Slightly more than half of the sites where we first recorded a dock in the 1960 decade were still occupied by a dock in the 2010 census decade (Table 3). Given the distribution of our imagery years (Table 1), this suggests that these sites have continually contained a dock for a minimum of 46–56 years, depending on the county. Because our imagery data began in the 1950s (Charleston or Colleton counties) or 1960s (Beaufort, Georgetown, Horry, and Jasper counties), sites that contained a dock in the first year of our analyses might have been occupied for even longer. Because we used the 2010 decade as our endpoint baseline year for determining dock longevity, the percentage of docks remaining reflects the percentage of dock sites in a decade that are still occupied in 2010. The percentage of docks removed is highest for docks built in earlier decades and for many cohorts of new docks there is a peak in the number of docks removed during the 1990 decade (Table 3).

**Table 3:** Table summarizing the longevity of dock presence at a site across all six coastal counties (excluding the 11 military/industrial tracts).

Decade	Number of new docks	Removed by 1960	Removed by 1970	Removed by 1980	Removed by 1990	Removed by 2000	Removed by 2010	Number remaining (2010)	Percent remaining (2010)
1950	197*	44	23	27	39	0	5	59	29.95
1960	792^†^	–	125	102	121	12	19	413	52.15
1970	676	–	–	46	123	12	10	485	71.75
1980	736	–	–	–	142	7	28	559	75.95
1990	3232	–	–	–	–	38	62	3132	96.91
2000	1691	–	–	–	–	–	27	1664	98.40
2010	3780	–	–	–	–	–	–	3780	100

The table should be read horizontally (e.g., of the 676 docks first identified in 1970, 46 were removed by the 1980 decade, 123 by the 1990 decade, etc.). Note that the “Number remaining (2010)” and “Percent remaining (2010)” columns do not suggest that the same docks have existed unrepaired or unaltered since they were constructed. These columns report the number/percentage of sites that have continuously contained a dock since one was first identified.

*As in Table 2, only Charleston and Colleton are represented in 1950.

^†^Unlike in Table 2, we report all docks first identified in 1960 for all counties. Since it is possible docks in Beaufort, Georgetown, Horry, and Jasper existed prior to 1960, these might not truly be “new” docks under our definition and should be understood as a minimum longevity in those counties.

### Objective 2—dock lengths

**Figure 3:**
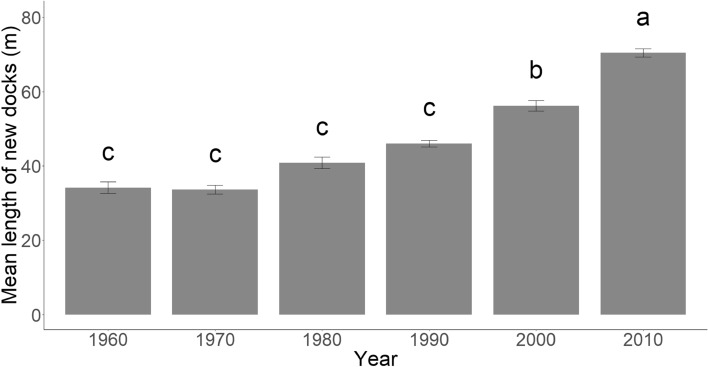
Results from the ANOVA of mean length of new docks over time (F_5,10684_) = 96.28, *p* < 0.001. Error bars represent standard errors. Different letters represent differences with a *p*-value < 0.05 between groups from post-hoc Tukey test (with p-values adjusted for multiple comparisons using a Bonferroni correction). Note again that 1960 only contains data from Charleston and Colleton counties.

Existing dock length demonstrated sizeable heterogeneity within all study years (Supplementary Fig. S6), although mean existing dock length increased by 19.46 m from the 1960s to 2010s (Table 4). As with dock counts, existing dock lengths were primarily driven by new construction. Mean new dock length roughly doubled from 34.13 to 70.38 m from the 1960s to 2010s. Our ANOVA showed a significant change in mean new dock length across the study duration (F_5,10684_ = 96.28, *p* < 0.001). A post-hoc Tukey test showed significant differences in new dock lengths in the 2000 and 2010 decades (Fig. 3). As noted earlier, the polygon format of the 2016 data prevented a simple straight-line measurement of dock length and we did not include it in the analysis.

**Table 4:** Table summarizing dock lengths across the study area (excluding the 11 military/industrial tracts).

Decade	New docks mean ± SD length (m)	Removed docks mean ± SD length (m)	Existing docks mean ± SD length (m)	Total linear km of existing docks
1950	–	–	49.07* ± 50.44*	9.67
1960	34.13 ± 31.65^†^	47.08 ± 44.82^†^	36.00 ± 35.54	34.02
1970	33.64 ± 30.53	34.43 ± 41.66	35.08 ± 32.61	51.67
1980	40.84 ± 42.87	42.44 ± 42.77	36.53 ± 35.74	74.30
1990	46.04 ± 50.93	40.2 ± 39.64	42.61 ± 46.51	206.28
2000	56.17 ± 57.99	47.21 ± 50.26	46.18 ± 50.11	298.48
2010	70.38 ± 67.89	44.03 ± 45.44	55.46 ± 58.76	559.88

New refers to docks we could verify were built in that decade, removed refers to docks that disappeared in that decade, existing refers to all docks during that decade.

*Only Charleston and Colleton had imagery in the 1950s and Colleton contained no docks. †Because 1960 is the first decade of imagery for Beaufort, Georgetown, Horry, and Jasper, these two columns in 1960 only represent Charleston and Colleton. The “Existing” column for 1960 represents all counties. After 1960 all columns report all counties.

### Objective 3—socioeconomic analysis of dock distribution

**Figure 4:**
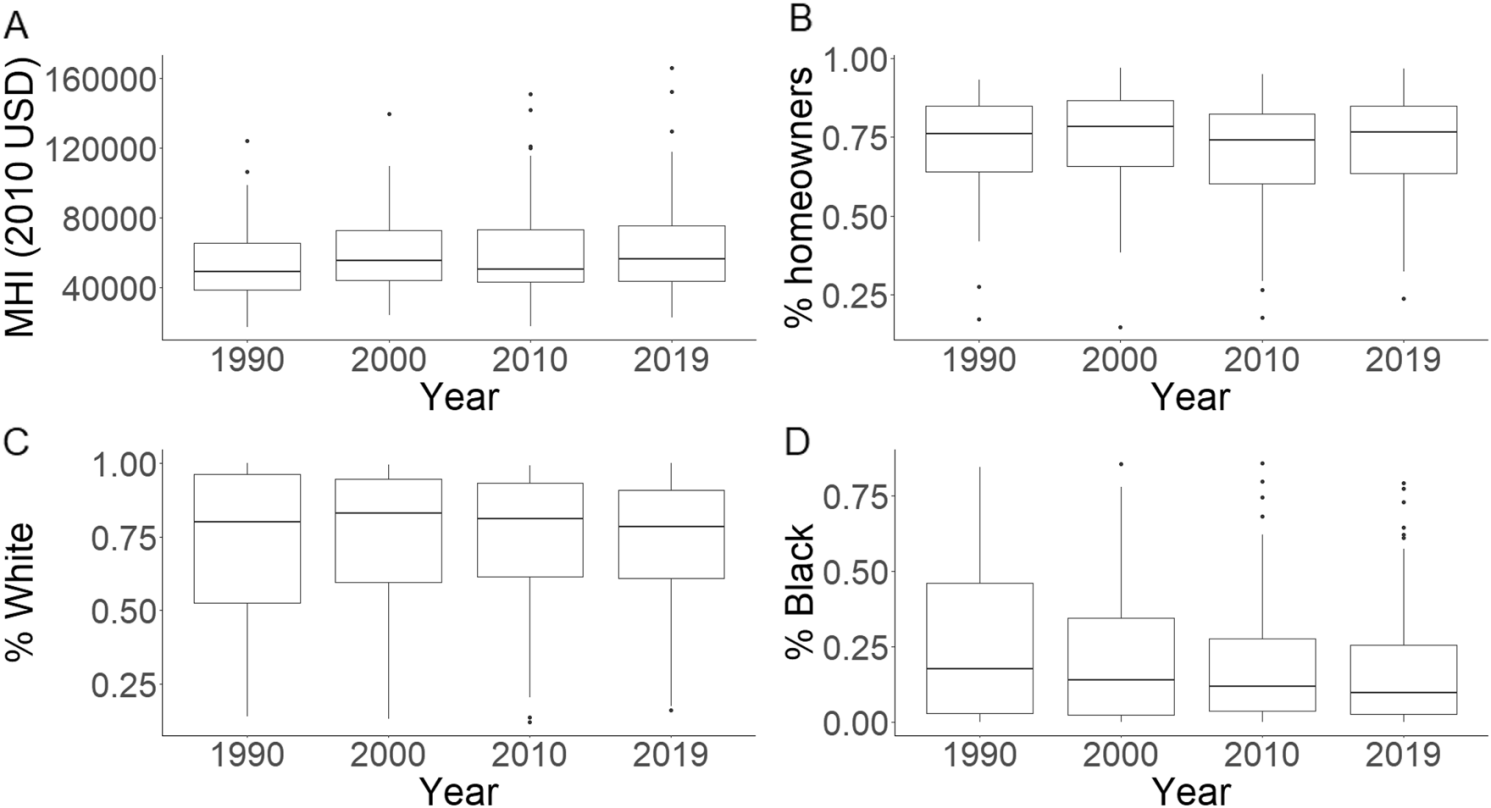
Boxplots of median household income (MHI, 2010 USD) (**A**), % homeownership (**B**), % White (**C**), and % Black (**D**) across the 111 tracts used in objective 3. The black midline of the boxplot represents the median, box edges represent the 25th and 75th percentile (interquartile range, IQR), error bars (also known as the upper and lower fence) represent 1.5 * IQR, and individual points represent outliers outside of the error bar range.

We conducted our generalized linear mixed models on the 111 tracts with greater than 1 km of saltwater shoreline. At the tract-level, median MHI (all values corrected to 2010 USD) fluctuated slightly across years but stayed relatively constant, varying from $49,105 in 1990 to $56,488 in 2019 (Fig. 4). The highest and lowest income tracts, however, varied more substantially. The lowest income tracts fluctuated through time, with a minimum MHI of $17,505 in 1990 and a maximum of $24,352 in 2000. The MHI of the wealthiest tracts increased consistently across the 4 decades, with a minimum MHI of $124,039 in 1990 to a maximum of $165,533 in 2019. Percent homeownership was steady through time, with the median varying between 73.9 and 78.4% from 1990 to 2019 (Fig. 4). The racial composition of tracts varied through time (Fig. 4). The median, minimum, and maximum percentage White residents remained consistent, hovering between 78.4 –82.9%, 12.1–16.1%, and 98.9–100%, respectively. The median percentage of the Black population fell through time, from 17.7% in 1990 to 9.9% in 2019. The maximum percentage of the Black population was steadier, ranging from 79.2 to 85.8% over time.

**Figure 5:**
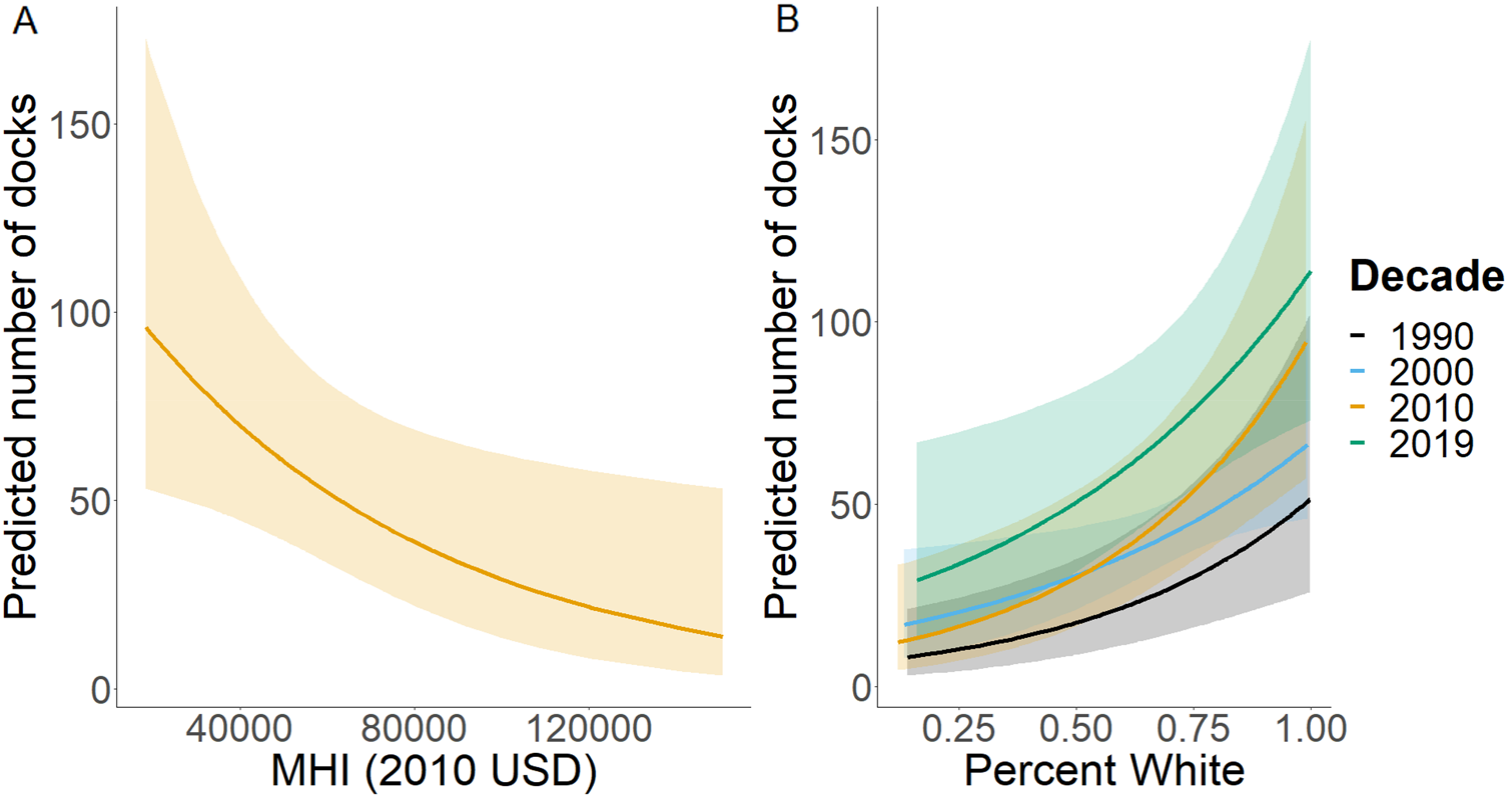
Conditional effects plots for all years of effects with a *p*-value < 0.05 for median household income (MHI) (**A**) and % White (**B**) on the predicted number of docks. Lines represent conditional effect predictions with corresponding bands representing 95% confidence intervals.

Our analysis revealed a consistently strong, positive relationship between percentage White and the abundance of private docks in each decade of data (Table 5, *p* < 0.05 for all likelihood ratio tests). On the other hand, MHI demonstrated a significant, negative relationship, but only in 2010 (Table 5, χ2(1) = 4.61, *p* = 0.03). There was a marginally significant, positive correlation between MHI and dock abundance in 2000 (Table 5, χ2(1) = 3.54, *p* = 0.06). As expected, percentage homeownership (except for the 2000 decade), population, and length of shoreline also showed significant, positive correlations with the number of docks in a tract (Table 5). The standardization of coefficients allows us to directly compare their magnitudes and therefore their relative strength on the abundance of docks. Length of shoreline is consistently the strongest effect, while percentage White, percentage homeowner, and total population had generally similar coefficient estimates (Table 5). The conditional effects plots generated from “ggeffects” (Fig. 5) contextualize these coefficients in more relatable numbers. For example, in 2000, the predicted number of docks was expected to increase from 17 to 65 in tracts moving from 0 to 100% White residents. Overall, these conditional effect estimates show a 6.4 × , 3.8 × , 7.8 × , and 3.9 × increase in dock abundance across the percentage White range (i.e., 0 to 100%) in 1994, 1999, 2011, and 2016, respectively. We provide complete model results, including random effects for tract and county in Supplementary Table S3.

**Table 5:** Table showing standardized regression coefficients for fixed effects from each decade.

Decade	Median household income	% White	% Homeowner	Population	Shoreline
1990	− 0.047	**0.54**	**0.52**	**0.27**	**0.66**
2000	0.32	**0.35**	0.19	**0.39**	**0.76**
2010	− **0.35**	**0.49**	**0.52**	**0.29**	**0.6**
2019	− 0.15	**0.35**	**0.40**	**0.33**	**0.61**

Values represent standardized beta coefficients from each decade and we bold terms with a *p*-value < 0.05 for identification. We provide full model results in Supplementary Table S3.

## Discussion

### Dock abundance and lengths

Our analysis of dock abundance and length conformed to our predictions, with both increasing over time. Considering the longstanding growth of the southern US in general^51–53^ and the coastal Southeast in particular^36,54,55^, the rising number of docks is unsurprising. Although dock abundance always increased (Table 2), there was a general oscillation between high (greater than 50% change) and modest (17–38% change) growth throughout our study years. Furthermore, in the periods of intense growth, docks increased at a much greater rate than either the number of housing units or population (Fig. 2). This is particularly apparent between the key inflection period of 1985–1994, when the number of existing docks in the coastal counties more than doubled. Interestingly, there appears to be a mismatch between periods of high dock growth and high population or housing unit growth (Table 2), suggesting that factors other than pure demography influence the timing of widespread dock development. Dock abundance might be showing signs of saturation following the 2010 decade (Fig. 2), but it is unclear if this is due to fewer available parcels of waterfront land, a general slowdown in development, or potential changes to the permitting regime in South Carolina making it more difficult to build docks. Regardless, docks have clearly demonstrated a sharp rate of increase that has outpaced both population and housing growth throughout the past 60–70 years (Fig. 2).

Our analysis found that 52% of sites that contained a dock built in the 1960 decade still contained a dock by 2011 (Table 3), strongly implying continuous dock presence for a minimum of 46–56 years. Even though these structures were undoubtedly repaired or even replaced during that time, these data suggest that once placed, docks frequently occupy a site for decades. The relatively small number of docks that were removed each decade was punctuated by a spike of 425 docks between 1985 and 1994. This spike in removals occurred regardless of when the dock had been constructed (Table 3). We suggest this peak is most likely a result of Hurricane Hugo, a destructive category four storm that made direct landfall on Sullivan’s Island (~ 7 km southeast of downtown Charleston, SC) in September 1989^56,57^.

Existing dock lengths also increased through time, primarily driven by the doubling of mean new dock length from 34.13 m in the 1960 decade to 70.38 m in the 2010 decade. Our ANOVA revealed that despite the wide variability in dock lengths, docks built in the 2000 and 2010 decade were significantly longer than docks built in prior decades (Fig. 3). Extremely long docks also became more prevalent in later decades, as evidenced by the increasing density of upper outliers in Supplementary Fig. S6. We believe this increase in length is likely a result of new construction occurring on parcels located further from deep water, as lots immediately adjacent to deep water are highly desirable and likely to be bought up first. Thus, newer parcels built along the marsh platform would necessitate longer docks to reach deep water.

All told, the steep rise in the abundance of docks, particularly from the 1990s onwards, along with the increasing length of new dock construction over the 2000 and 2010 decade, indicate a growing desire among coastal residents for direct, private access to estuaries. Cumulatively, docks covered 559.88 linear km of South Carolina’s coastline by the 2010 decade (Table 4), more than 3.5 times the 159.31 linear km of shore-parallel armoring infrastructure in the same region as of 2013^58^. Although important differences exist between the ecological and hydrological impacts of shore parallel infrastructure (e.g., armoring such as seawalls, bulkheads, and riprap) and shore perpendicular infrastructure (e.g., docks)^59^, the extent of private docks suggests that they may have considerable cumulative impacts on coastal ecosystems.

### Economic distribution of docks

Our analysis of the historic demographic distribution of docks partially aligned with our hypotheses. Contrary to our expectations, median household income showed an inconsistent correlation with dock abundance, both in terms of direction, statistical significance, and magnitude of effect. We found a significant, negative correlation in the 2010 decade and a borderline significant, positive correlation in the 2000 decade. Racial composition (percentage White) showed a strong positive correlation with dock abundance in all decades. There is, however, little evidence that racial composition and time interacted over our study period, as the coefficient estimates for the percentage White term in our models remain positive and stable.

The variability of the relationship of median household income is peculiar and difficult to parse with these data. Why household income shows weak and insignificant effects in 1994 and 2016 but shows significant (or nearly so) effects that switch signs between 1999 and 2011 could have several interacting explanations. First, we may be incorrectly conceptualizing the relationship between docks and personal income by ignoring important decisions made by homeowners. We predicted an increasing abundance of docks with higher income because docks are expensive structures to build and maintain. Docks, however, increase home value and are perceived as desirable additions by homeowners^60,61^. Modest income homeowners may tolerate the high initial costs of building a dock to increase the value of their home, leading to a dampened effect of income on dock abundance. Second, we might be missing important predictor variables that covary with MHI, such as the size of private property lots. It is feasible that the increasing disparity between the lowest and highest income tracts through time (Fig. 4) moves alongside a contemporaneous disparity in mean lot sizes, with higher MHI households having bigger lots. All else being equal, larger average lot sizes in a tract would result in fewer docks because individuals cannot build more than one dock per lot under current South Carolina regulations (S.C. Code of State Regulations §30–12-A-1a), potentially explaining the negative relationship between MHI and dock count in 2011. Unfortunately, we were unable to acquire data on lot sizes because many municipalities that possess this information did not respond to our requests.

Third, median household income is only one measurement of economic status and other variables, such as the availability of liquid assets or length of tenure in a home, might be more reflective of an individual’s desire, ability, and willingness to pay for a dock. Lastly, as we discussed in our earlier work^9^, the inconsistent relationship between income and dock abundance might be a consequence of the limitations imposed by aggregated spatial data that cannot capture the effect of income at the household level^22^. Prior survey work in Alabama, Florida, and North Carolina found that waterfront property owners tended to be wealthier^62,63^ and Whiter^63^ than their inland neighbors. Ultimately, we believe a combination of housing development drivers occurring at a spatial scale below the tract level are the likely root of these fluctuating income relationships. Future work at the household level would be needed to test these potential explanations. The specificity that surveys and interviews could provide would be valuable in disentangling the relationship between docks, waterfront property ownership, and personal wealth.

### Racial distribution of docks

We found a persistent racial imbalance in the distribution of private docks from 1994 to 2016, with tracts that contained a larger proportion of White residents possessing substantially more docks. Our imagery sets allowed us to assess general development patterns as far back as the late 1940s-1950s for most counties, but we were unable to use this full data set for our analysis of the socioeconomic distribution of docks. This is an unfortunate consequence of working with historical census data in rural areas, as they generally did not contain spatial units at the sub-county level until the entire United States was divided into tracts for the 1990 census^40^. Despite the limitations imposed by working with relatively coarse tract-level data, this study provides valuable insights into the longevity of environmental injustices around estuary access that exist in South Carolina. Furthermore, our analysis of the 2016 data resulted in the same qualitative patterns whether conducted at the tract-level (this study) or finer-grain census block group level^9^, suggesting robustness to differing levels of spatial aggregation^64^.

Although observational studies cannot directly assign causality, we offer three mechanisms that could explain the positive correlation between the percentage of a tract that identifies as White and dock abundance:

1. History of property ownership
2. Aggregation obscures an interaction of race and income
3. Bias in dock permitting

The first mechanism is rooted in historical analyses of property ownership along the Southeast coast. Following the end of the Civil War and the collapse of the plantation economy, the perceived marginal value of coastal land enabled emancipated Black and Gullah-Geechee communities to acquire tremendous amount of property throughout the coastal Southeast^65–67^. There have been, however, numerous obstacles to holding onto that property. The tenuous nature of Black landownership in this region stems from multiple drivers, including predatory practices by real estate developers^68,69^, the threat of forced partition sales due to heirs property status^70–72^, and property tax policies that disproportionately burden Black landowners^68,73,74^. When coupled with rapidly appreciating values of waterfront land, these forces helped fuel the well documented, precipitous decline of Black landownership throughout the mid-twentieth century^65,75,76^. As Black landowners lost (and continue to lose) their land, these properties were characteristically bought up and converted into sprawling planned communities catering to predominantly White internal migrants from other parts of the US^77,78^. This dual process of Black displacement and ensuing subdivision of properties into multiple parcels could produce the positive correlation we observed between dock abundance and the proportion of a tract’s population that is White. We noted some support for this proposed mechanism in our data. The median percentage Black population of our coastline tracts halved from 1990 to 2019, while the median percentage White population remained stable. This pattern suggests that Black residents are being displaced from coastal tracts, while White residents maintain their share of the population. Furthermore, even after controlling for the length of shoreline, predominantly White tracts consistently possessed more docks across all decades, suggesting that the way the shoreline is divided among racial groups could be an important factor. Concrete evidence in support of this mechanism could come from a deep analysis of property records, with a focus on how parcel size has changed over time. Some historians have undertaken this work in other Southern states^73,75^, but we know of no robust analysis of property transfers and parcelization in our counties.

Our second mechanism draws from our discussion above about potential mismatches between income aggregated at the tract level and income at the household level. If White homeowners have more disposable financial resources and aggregation of household income obscures this, then that could explain the observed positive correlation between percentage White and dock abundance, as well as the inconsistent correlation between MHI and dock abundance. Ample evidence of a national disparity in household wealth between Black and White Americans lends credence to this proposed mechanism^79^. Like Mechanism 1, there is some evidence in our data to support this mechanism. We quantified a modest correlation between percentage White and MHI (Supplementary Fig. S7) at the tract level in all years of the study. We note, however, that we tested for and found no interaction of race and median household income and that the correlation between these two variables did not produce any collinearity issues. Additionally, our previous analysis at the finer-grain census block group level similarly found no covariate interactions between race and income^9^. Testing this mechanism further would require finer disaggregation of our response variables to as close to the household level as possible (e.g., the census block). Future data releases, such as the recent publishing of complete 1950 census records, might alleviate these limitations, though working through these handwritten records requires considerable effort and full census records are not released until 72 years after they are collected.

Our third mechanism posits that bias in dock permitting drives the racial imbalance in dock abundance. South Carolina did not require permitting for dock construction until the passage of the Coastal Tidelands and Wetlands Act in 1976 (S.C. Code Ann. §48–39 *et seq.*), which itself was passed to implement the requirements of the federal Coastal Zone Management Act of 1972 (Public Law 92–583, 16 U.S.C. §1451 *et seq.*). If systematic differences in the approval of docks from White and non-White applicants occurred historically or is currently occurring, then White areas may contain more docks. There is little literature examining the history of dock permitting in South Carolina and we have not identified any work examining racial bias in permitting decisions for coastal infrastructure in the state. Unfortunately, we also know of no comprehensive historical data on permitting decisions made by the Ocean and Coastal Resource Management (OCRM) division of the South Carolina Department of Health and Environmental Control. The vast number of docks in South Carolina suggests that the state’s permitting system has not been a significant barrier to dock construction. Although comprehensive data on dock permitting is not publicly available, the little information we are able to piece together suggests that dock permit applications increased in the 1990s relative to the 1980s, which aligns with our data^80^. It also appears that very few dock permits are denied by OCRM each year. For example, in 2000 and 2001 OCRM approved 717 out of 725 and 808 out of 815 dock permit requests it received, respectively^81^. Taken together, this data suggests that, at least in contemporary times, systematic bias in dock permitting is a less likely explanation than Mechanism 1 or 2.

Regardless of the underlying mechanisms, we argue that the preponderance of docks in predominantly White tracts is an environmental justice issue that has persisted for at least two decades. While we are intentionally working under a narrow definition of access^4^ that prioritizes the capacity for physical entry into estuaries and salt marshes, the strength and consistency of the racial imbalance in dock abundance is clear from our data. Because docks both facilitate entry into waterbodies and are a conservative proxy for waterfront private property, our analyses suggest that White South Carolinians have possessed a greater share of waterfront properties and therefore greater access to estuaries than other groups in South Carolina during the 1990–2010s. We also argue that due to sub-tract level patterns of development that our data cannot capture, we are likely underestimating the degree to which docks and the waterfront property they occur on are concentrated in the hands of White South Carolinians. As noted earlier, survey work conducted along the Gulf Coast in Alabama and Florida found that waterfront property owners tended to be wealthier and Whiter than property owners in the surrounding area^63^. When paired with rich historical work demonstrating that the exploitation of Black landowners was a driving force in real estate and tourism development in the South^65,68,76^, we believe further quantitative and qualitative work is needed to distinguish between the alternative mechanisms we propose, as these point to potential avenues for reconciling the disparity in private access.

## Conclusion

Docks are long-lived structures that have grown at twice the rate of the population or housing units along the South Carolina coast since the 1960s. As of 2011, 10,092 docks, totaling 560 linear kilometers dotted the estuaries and major coastal rivers of six South Carolina counties, which is more than 75% greater than the nominal length of the state’s coastline (301 km). Although there is a wealth of research on the effects of anthropogenic structures on ecosystem productivity^38,39^, community composition^82–84^, and a range of other ecological processes^59,85,86^, recent attempts to quantify the extent of marine infrastructure have largely focused on large-scale, commercial^32^, or armoring infrastructure^29^. Our work suggests that small, private docks can be the most abundant and long-lived form of marine infrastructure in some regions and should be considered in subsequent analyses.

Apart from their ecological effects, docks both facilitate human activity in estuaries and mark the location and extent of privatized waterfront. We found that the distribution of docks, and therefore access to coastal waters, has skewed in favor of White South Carolinians since at least the early 1990s. Given the central importance of estuaries to communities throughout the Southeast US^87^, this racial imbalance in access constitutes an entrenched EJ issue. Our work, when placed in context with the insights of communities, historians, and geographers suggests that while the waterways of South Carolina are held as a public resource, a predominant means of accessing them remains inequitably distributed.

### Supplementary Information

Supplementary Video 1.

Supplementary Information 1.

## Data Availability

The datasets analyzed during the current study are available in the Dryad repository, https://doi.org/10.5061/dryad.fn2z34tzw. The 1944 and 1999 aerial imagery are available from the corresponding author on reasonable request. The pre-1990 aerial imagery is available from the University of South Carolina’s Thomas Cooper Library, but restrictions apply to the availability of these data, which were used under license for the current study, and so are not publicly available. Data are however available from the authors upon reasonable request and with permission of the Department of Government Information & Maps at the University of South Carolina’s Thomas Cooper Library. Imagery from 2011 is available from the United States Department of Agriculture National Agriculture Imagery Program’s Geospatial Data Gateway, (https://nrcs.app.box.com/v/gateway/folder/19350726983).
