# Supplementary material for: Exponential growth of private coastal infrastructure influenced by geography and race in South Carolina, USA
Source: Sci Rep. 2024 Apr 20;14:9114. doi: 10.1038/s41598-024-59740-x (PMC11032348; doi:10.1038/s41598-024-59740-x)
Supplement: Supplementary file 2 — Supplementary Information 1. [file 41598_2024_59740_MOESM2_ESM.docx]

**Supplementary Material to “Exponential growth of private coastal infrastructure influenced by geography and race in South Carolina, USA”**

**Preface**

This appendix provides further information on some of the methods used in the study. The raw data, and R scripts are available in the public data repository for this project: https://doi.org/10.5061/dryad.fn2z34tzw. Additionally, a full step-by-step guide is also available in the repository if you desire even further methodological detail. If you have any questions, please feel free to contact the corresponding author: Jeffrey Beauvais (he/him), beauvais.work@gmail.com.

**Structure Assignment Rules and Quality Control**

Pre-1990 image quality varied between 1.25-4.5 m resolution. A team of four undergraduate assistants and J. Beauvais checked the pre-1990 imagery. While in many instances the presence of docks was clear in the pre-1990 imagery, the image quality could sometimes impede object identification. To ensure consistency in deciding whether an object was a dock across varying image resolutions and five different researchers, we established a set of four criteria that had to be met for an object to be counted as a dock:

1. The object must have a base that is clearly attached to solid land
2. The object must terminate at or beyond the water’s edge
3. Must have physical qualities of human-built infrastructure (i.e., clean edges, no undulation along the length of the object)
4. Occur in a logical place and have a logical shape for a dock (i.e., it must be linear and approach water directly with at most one bend)

We trained all undergraduate assistants over at least two sessions and encouraged them to mark any structures they were unsure of. Following completion of a county/year imagery set, J. Beauvais checked all structures marked for additional review and made final decisions. J. Beauvais also conducted random spot checks to ensure that undergraduate researchers were evenly applying the listed criteria. In instances where different images from the same year overlapped in space or multiple images of the same with area with varying resolution occurred, we always relied on the higher quality resolution to make final decisions on whether an object was a dock or not.

Imagery from 1994, 1999, and 2011 were of sufficient resolution to allow for easy identification of docks in almost all cases and while the criteria listed above still applied, we did not have to deliberate whether individual objects in question met them because of the high quality. Additionally, only J. Beauvais collected data on the 1994, 1999, and 2011 imagery.

Our approach of sequentially building on prior GIS layers (e.g. the completed dock point layer for Beaufort 1955 served as the starting point for 1959, 1959 served as the starting point for 1972, etc.) necessitated criteria for determining which structures were new (Figure S1), removed (Figure S2), or maintained (Figure S1) due to variations in georeferencing. An example of these situations is provided in Figure S1. As noted in the main text, we relied on the attributes of the points, number of docks in the image, comparison between different years of imagery, and other context to make these decisions. For example, in the top image of Figure S1 we see the original scene in 1955, where we placed six sets of red points on the six docks (we have intentionally offset the points to avoid obscuring the docks for illustrative purposes). In the bottom image of Figure S1 we see the same area in 1959. In this scene, the points we placed in 1955 have shifted slightly downwards and to the right of the actual docks in the image. We needed to determine if these are the same docks in the 1955 and 1959 images. First, the shifts are minor and well within expectations for georeferencing accuracy of historic imagery. Second, the angle and distance between each set of points relative to the docks in the scene is consistent. Third, between the second and third dock (from the left) we see these docks in the same position on the shore relative to the concave section of coastline, which is clearly the same in the 1955 and 1959 images. Thus, we considered these six docks as being maintained from 1955 to 1959 and we marked three new docks (blue circles).

**Fig. S1** Top – 1955 photo with six sets of red points placed at the upland base and waterward end of the docks. Red points are shifted slightly left for ease of viewing each dock in the image. Bottom – 1959 photo with retained docks (red points) plus three new docks (blue circles). Note that in the bottom image the red points have shifted due to errors in georeferencing between imagery years. Image scales are 1:5,000. The figure was created from imagery provided by the University of South Carolina’s Thomas Cooper Library and is reprinted with their permission.


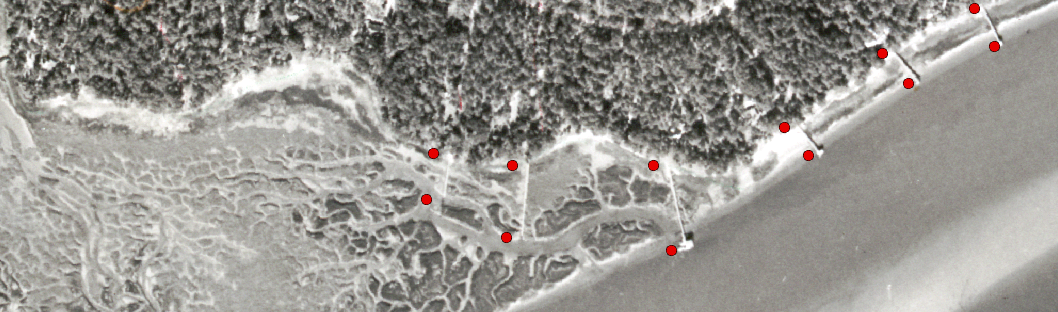

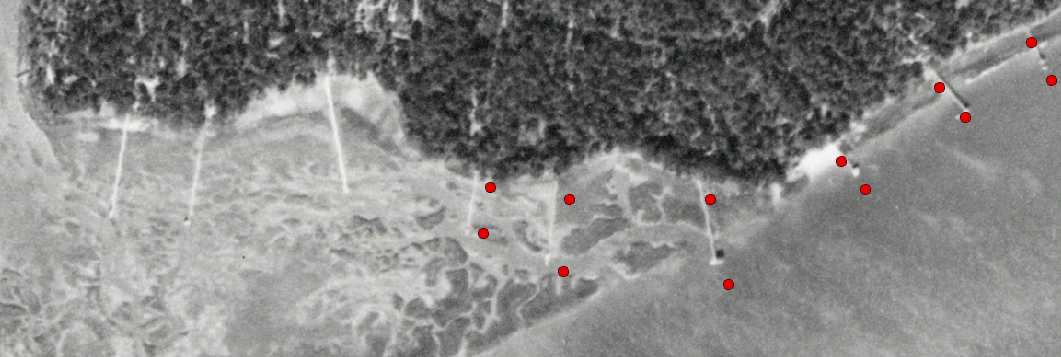


**Fig. S2** Left – a dock in 1955, Right – same area in 1959 showing the 1955 points (green) and no dock. It is clear the dock was removed in the intervening years. Image scales are 1:1,600. The figure was created from imagery provided by the University of South Carolina’s Thomas Cooper Library and is reprinted with their permission.


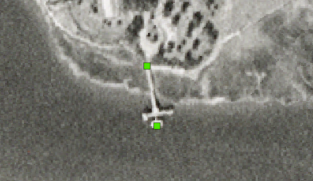

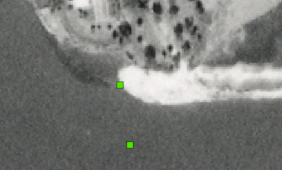


**Identifying public infrastructure**

It was not possible from imagery alone to definitively know whether a dock was publicly or privately owned. Publicly owned structures, such as public fishing piers and mooring docks located at public boat landings, are often visually indistinguishable from a privately owned dock. An underlying assumption of our work is that docks are a conservative proxy for privately owned, waterfront property. Therefore, if we incorrectly labeled public owned dock as privately owned, our assumptions would be violated. To ensure minimal violations of this assumption, we took steps to identify and omit public infrastructure from our study. First, we used context clues to infer and mark docks that we thought might be publicly owned. The presence of a large adjacent parking lot, numerous cars with boat trailers, or a dock immediately adjacent to a bridge are often good signs that the dock is publicly owned. Whenever we encountered a dock we suspected might be publicly owned, we marked it as a tentative public structure and made a note to review it. A full list of these structures is provided in the “Public WAI” tab of the “Historic Mapping Work Log” Excel document, which is in the “Historic_Dock_Supplemental” folder in the Dryad repository (link provided at the top of this document).

Once we completed our search of the imagery, we contacted local and county governments for records on when they built public fishing piers and boat landings. Unfortunately, all officials informed us that records on **when** these structures were built were functionally nonexistent if we went back further than the early 2000s. That said, we did have 2016 data on public infrastructure from our prior publication (Beauvais et al. 2022), as well as local, county, and state government websites listing all the public fishing piers and boat landings in our counties. We decided to cross-reference our data set with this information to see if a structure we **suspected** was publicly owned from **historic** imagery was publicly owned as of 2016. In total, we marked 63 structures we suspected were publicly owned (34 suspected public fishing piers and 29 suspected mooring docks located at public boat landings). Of those, we could definitively determine that 59 of the 63 structures were publicly owned as of 2016 (31 out of 34 public fishing piers and 28 out of 29 mooring docks located at public boat landings). We decided to omit all 63 of these structures from the study.

As of 2016, we could identify 120 publicly owned docks in our study area (47 public fishing piers and 73 mooring docks located at public boat landings). After removing the 59 structures discussed above (as well as the additional 4 we could not verify were publicly owned), this leaves 61 publicly owned structures (as of 2016) that we did not identify when reviewing the historical imagery. This results in a spectrum of possibilities, with one extreme being that all 61 of these structures were built between 2011 and 2016 (and therefore we did not incorrectly label any structures as privately owned), and the other extreme being that all 61 of these structures have existed since the 1950s/1960s (and therefore all 61 structures are incorrectly labeled as privately owned). Therefore, we incorrectly assumed that somewhere between 0-61 publicly owned docks are privately owned. Given the number of docks we identified within each decade, this means that at worst we overestimated the number of privately owned docks in the 1960 decade by 6.5% (61/945, Table 1, main paper), which reduces to 0.60% in the 2010 decade (61/10,092). We believe these are acceptable error ranges given the limitations of our data.

**Imagery bins**

Binning our imagery was necessary to pair our dock counts with data from the census, which occurs every 10 years. Given the temporal distribution of our imagery, some imagery bins contained multiple corresponding years of imagery for a single census (Table 1, main paper). We argue this is a minor issue. First, due to the limitations of extrapolating data in areas that did not historically contain tracts, we do not conduct any tract-level statistical analyses prior to 1990 at which point there are no duplicate years of imagery in a bin (Table 1, “All counties”). Second, the major potential issue of increased resolution within imagery bins is if a dock was first identified and removed within the same census decade (e.g. a dock first identified in Beaufort 1955 that is removed in 1959, Table 1). This situation only occurred to 14 structures in the 1960 census decade, and we have removed them from the analysis.

**Temporal county level data**

We present a GIF detailing dock locations over the study period in Figure S3 and Online Resource 1. We also present counts and lengths of new, removed, and existing docks at the county-level for those interested in an even more fine-grain look at dock development in Figures S4 and S5. Charleston dominated in dock abundance throughout the entire study period, followed by Beaufort. Lastly, we provide a boxplot in Figure S6 to demonstrate the heterogeneity in existing dock lengths. Although we did not conduct any statistical analyses, a qualitative look suggests that dock lengths vary by county, with Beaufort, Charleston, and Jasper counties having longer docks on average than the other counties. Horry County consistently contains the shortest average dock length. We believe differences in dock sizes by county might be rooted in landscape characteristics such as channel morphology and marsh depth across the counties. For example, Horry County contains very little open marsh and most docks are built along a narrow, channelized portion of the Intracoastal Waterway, allowing docks to reach the water in a shorter distance. This would also likely require them to be shorter in length so they do not impede navigation on the Intracoastal Waterway.


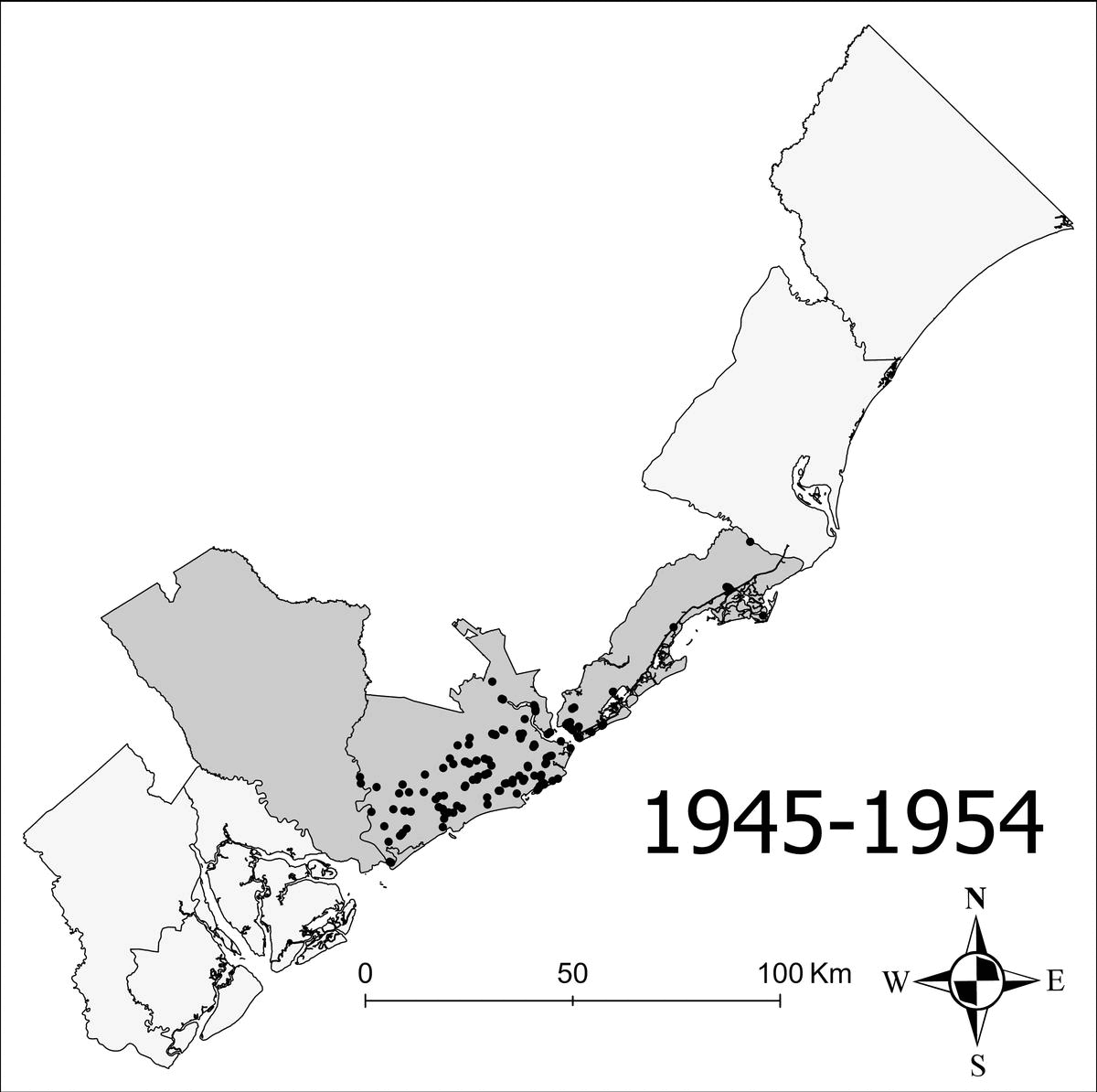
**Fig. S3** Existing docks across the study area over all decades in the study. File is a GIF which cycles through all decades sequentially. Areas without imagery in the 1945-1954 period are a lighter shade of gray. Circular points represent existing docks, while red triangles represent docks that were removed in that period. Removed docks are subsequently removed from future decades. Circular points are colored based on the decade they first appeared. Black circles – docks added from 1945-1954, orange circles – docks added from 1955-1964, light blue circles – docks added from 1965-1974, green circles – docks added from 1975-1984, yellow circles – docks added from 1985-1994, dark blue circles – docks added from 1995-1999, white circles – docks added from 2000-2011. Image scale is 1:1,225,000. The figure was created in ArcGIS Pro Version 2.9. 2010 County boundaries were downloaded from the National Historical GIS data selector (https://data2.nhgis.org/main).


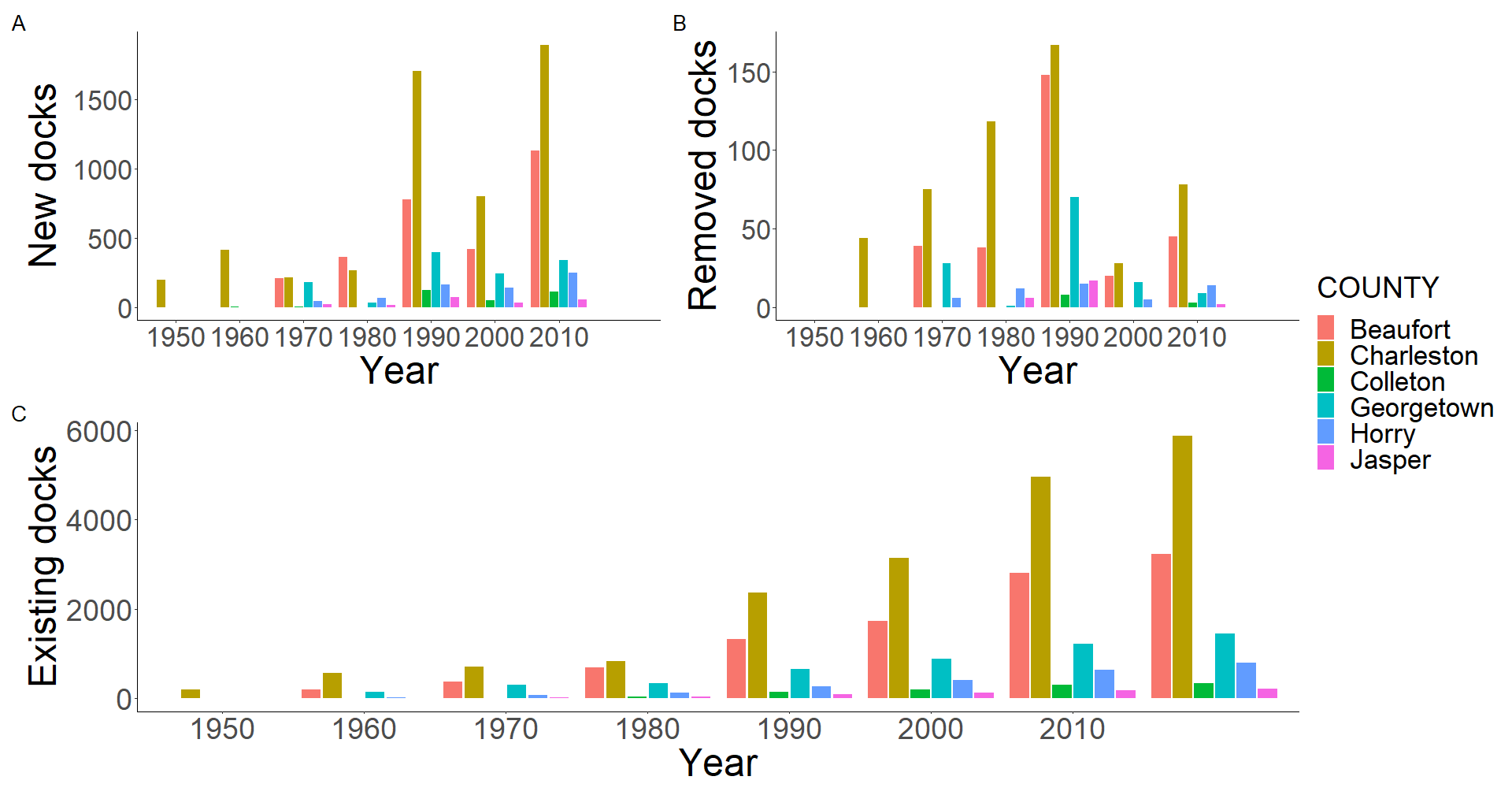
**Fig. S4** County level dock counts from 1950-2019. Note that only Charleston and Colleton counties contained imagery from the 1950 decade and Colleton County contained no discernible docks at this time. Also, all counties contained docks in 1960, but Colleton (2), Horry (21), and Jasper (7) counties are difficult to see given the scale of the y-axis.


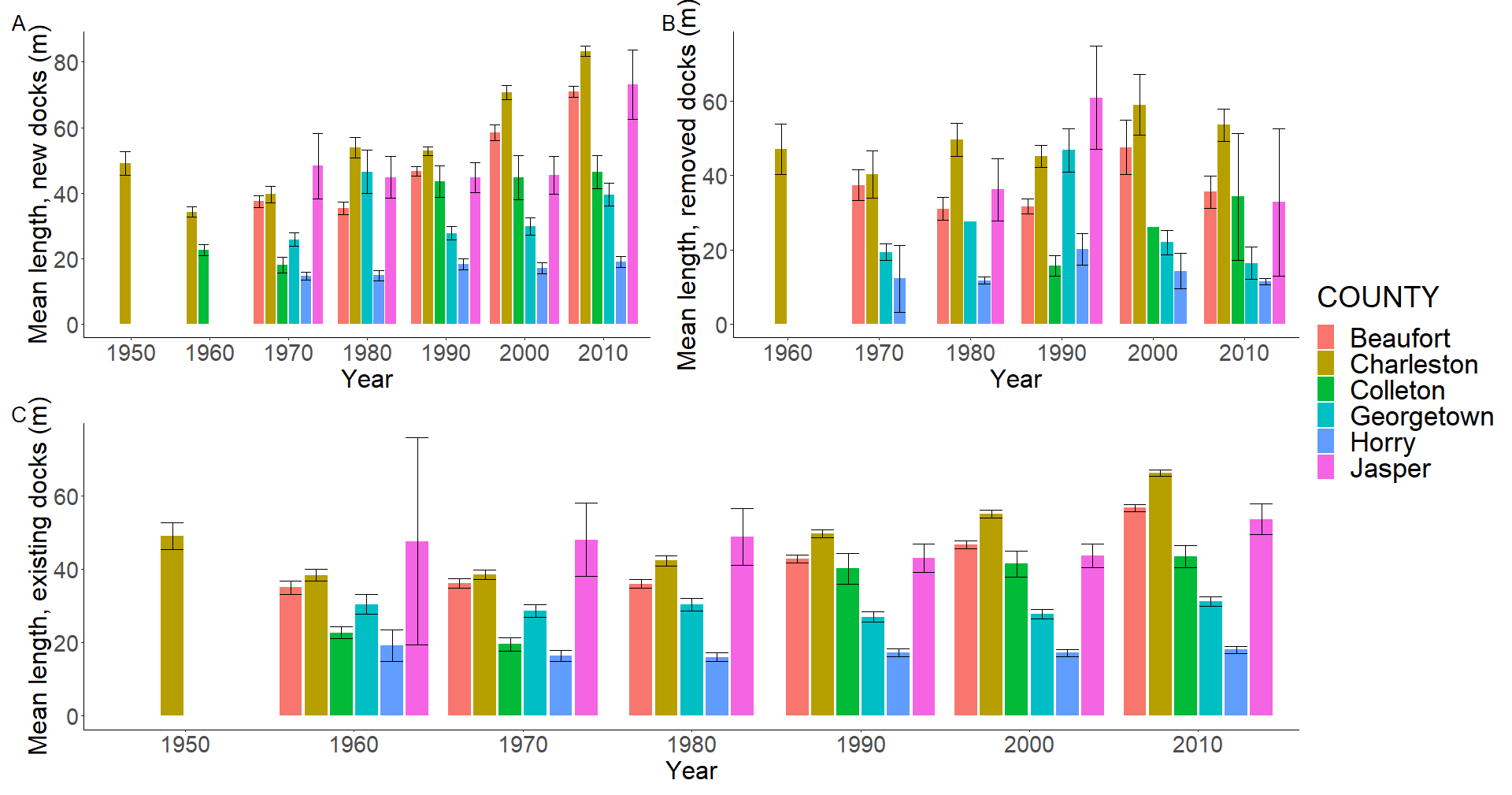
**Fig. S5** County-level mean dock lengths for new, removed, and existing docks. Note that only Charleston and Colleton counties contained imagery from the 1950 decade and Colleton County contained no discernible docks at this time. Error bars represent standard errors.


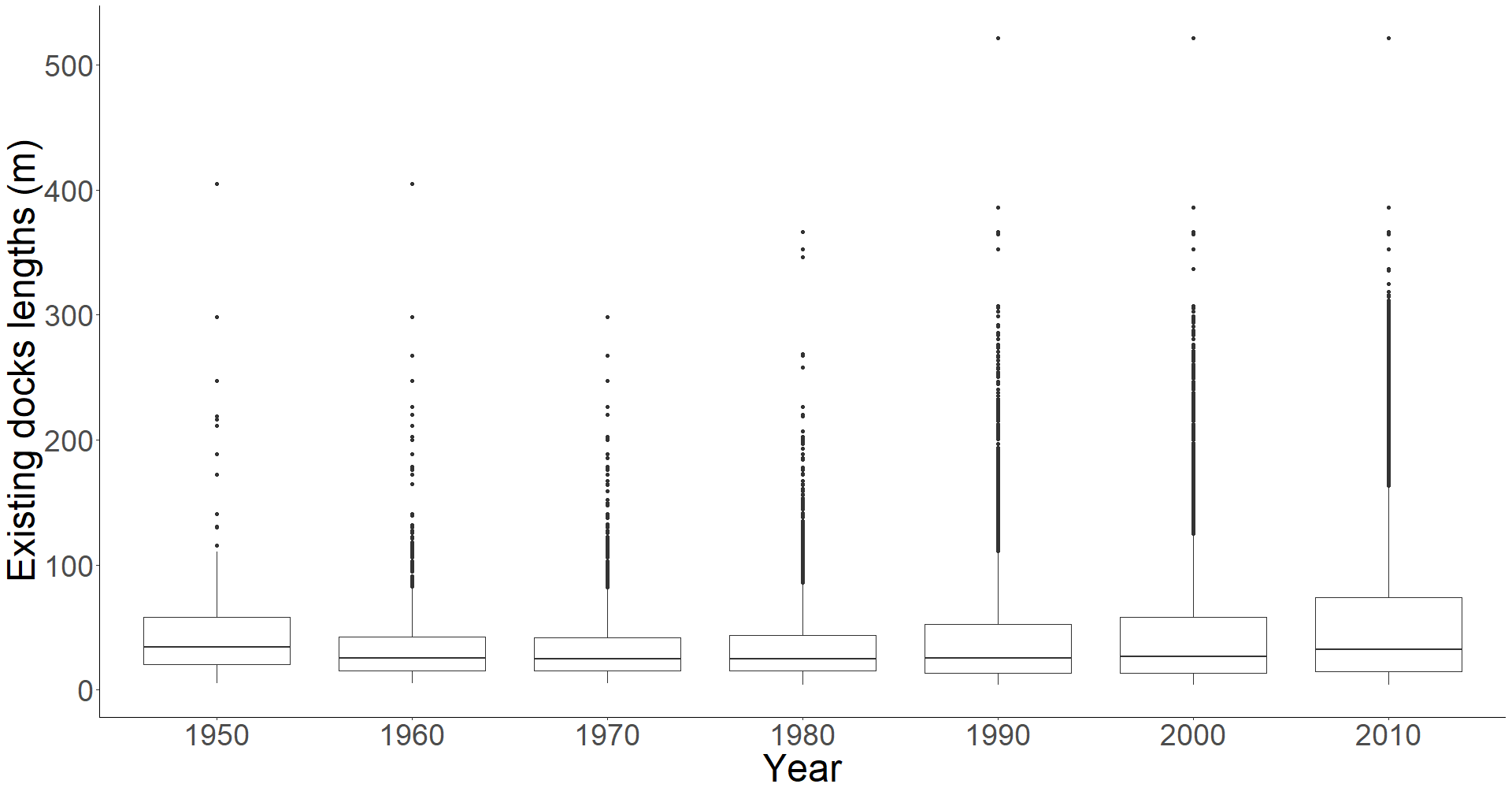
**Fig. S6** Boxplot of existing dock lengths across the study area. The black midline of the boxplot represents the median, box edges represent the 25^th^ and 75^th^ percentile (interquartile range, IQR), error bars (also known as the upper and lower fence) represent 1.5 * IQR, and individual points represent outliers outside of the error bar range.

**Shore Length and Housing Unit Cutoff**

We derived our shore length estimates from a prior study completed (Beauvais et al. 2022) using 30-m resolution land cover data from NOAA’s Digital Coast Portal ([https://coast.noaa.gov/digitalcoast/data/](about:blank)). We decided to have a minimum shoreline requirement for inclusion in Objective 3 because we wanted to make sure that tracts had an appreciable amount of shoreline that would feasibly allow docks. We chose to be more conservative with our measurements because the relatively low resolution of the 2016 C-CAP NOAA land cover data used to measure shoreline can cause areas of marsh to be incorrectly classified as developable land, artificially inflating shore length. We also instituted a cutoff based on the number of housing units in a tract to avoid potential issues that can occur in estimating demographic characteristics of very sparsely populated areas. We opted to use a 1 km shoreline cutoff (to be consistent with the prior work) and a 100 housing unit cutoff. We recognize that these cutoff values are arbitrary, so we have included the tables below (Tables S1 and S2) that quantify how many tracts and docks are lost based on different cutoff levels for the length of shoreline or number of housing units. The 1 km shoreline cutoff had a slight effect, with a maximum of 586 docks (or 4.9% of existing docks) excluded in the 2019 decade. The choice of housing unit cutoff had essentially no effect, as only four tracts with 35 existing docks (or 0.7% of existing docks) were excluded in 1990. Note that for the 1 km cutoffs we also remove one tract in Georgetown County that was an extreme outlier in the ratio of freshwater to saltwater shoreline (193 km of freshwater shoreline and 1.02 km of saltwater shoreline). Although this does technically meet the 1 km threshold, it was by far the largest outlier and we felt it was inappropriate to include. 12 other tracts had more freshwater than saltwater, 5 of which had > 5 km of freshwater shoreline. We kept these tracts because the ratio of freshwater to saltwater was much more reasonable.

**Table S1** Table showing the number of tracts and docks included for four different levels of minimum shoreline length requirements. The “% of docks” column documents what percentage of docks remain relative to no cutoff.

|  | **Cutoff (km of shoreline)** | | | | | | | | | | | | | | |
| --- | --- | --- | --- | --- | --- | --- | --- | --- | --- | --- | --- | --- | --- | --- | --- |
|  | 0 | | | 0.25 | | | 0.5 | | | 0.75 | | | 1 | | |
| **Year** | # of tracts | # of docks | % of docks | # of tracts | # of docks | % of docks | # of tracts | # of docks | % of docks | # of tracts | # of docks | % of docks | # of tracts | # of docks | % of docks |
| 1990 | 212 | 4841 | — | 120 | 4712 | 97.3 | 119 | 4712 | 97.3 | 116 | 4711 | 97.3 | 111 | 4670 | 96.5 |
| 2000 | 212 | 6463 | — | 120 | 6243 | 96.6 | 119 | 6243 | 96.6 | 116 | 6242 | 96.6 | 111 | 6182 | 95.7 |
| 2011 | 212 | 10092 | — | 120 | 9701 | 96.1 | 119 | 9701 | 96.1 | 116 | 9694 | 96.1 | 111 | 9599 | 95.1 |
| 2019 | 212 | 11886 | — | 120 | 11445 | 96.3 | 119 | 11445 | 96.3 | 116 | 11432 | 96.2 | 111 | 11300 | 95.1 |

**Table S2** Table showing the number of tracts and docks included for four different levels of minimum number of housing units. This table builds from Table S1, using the 1 km shoreline filter we decided upon as the starting point (i.e. the value for when the cutoff for housing units is 0). The “% of docks” column documents what percentage of docks remain relative to a 1 km shoreline cutoff and no housing unit cutoff.

|  | **Cutoff (number of housing units)** | | | | | | | | | | | |
| --- | --- | --- | --- | --- | --- | --- | --- | --- | --- | --- | --- | --- |
|  | 0 | | | 50 | | | 75 | | | 100 | | |
| **Year** | # of tracts | # of docks | % of docks | # of tracts | # of docks | % of docks | # of tracts | # of docks | % of docks | # of tracts | # of docks | % of docks |
| 1990 | 111 | 4670 | — | 108 | 4635 | 99.3 | 108 | 4635 | 99.3 | 107 | 4635 | 99.3 |
| 2000 | 111 | 6182 | — | 110 | 6182 | 100 | 110 | 6182 | 100 | 109 | 6182 | 100 |
| 2011 | 111 | 9599 | — | 111 | 9599 | 100 | 111 | 9599 | 100 | 111 | 9599 | 100 |
| 2019 | 111 | 11300 | — | 111 | 11300 | 100 | 111 | 11300 | 100 | 111 | 11300 | 100 |

**Table S3** Regression output for fixed and random effects from each decade. Significant p-values are bolded for identification.

* indicates that the likelihood ratio test could not converge for the reduced model – this represents the Wald estimate.

| **Variable** | **Decade** | **Coefficient Estimate** | **SE (fixed)/Variance (random)** | **LRT Result** |
| --- | --- | --- | --- | --- |
| MHI (income) | 1990 | -0.047 | 0.14 | χ2(1) = 0.10, p = 0.74 |
|  | 2000 | 0.32 | 0.15 | χ2(1) = 3.54, p = 0.06 |
|  | 2010 | -0.25 | 0.16 | χ2(1) = 4.61, **p = 0.03** |
|  | 2019 | -0.15 | 0.16 | χ2(1) = 0.70, p = 0.40 |
| % White | 1990 | 0.54 | 0.16 | χ2(1) = 11.89, **p < 0.001** |
|  | 2000 | 0.35 | 0.14 | χ2(1) = 5.78, **p = 0.02** |
|  | 2010 | 0.49 | 0.14 | χ2(1) = 10.77, **p = 0.001** |
|  | 2019 | 0.35 | 0.14 | χ2(1) = 5.43, **p = 0.02** |
| % Homeowners | 1990 | 0.52 | 0.15 | χ2(1) = 11.23, **p < 0.001** |
|  | 2000 | 0.19 | 0.15 | χ2(1) = 1.48, p = 0.22 |
|  | 2010 | 0.52 | 0.14 | χ2(1) = 11.69, **p < 0.001** |
|  | 2019 | 0.4 | 0.13 | χ2(1) = 7.78, **p = 0.005** |
| Population | 1990 | 0.27 | 0.11 | χ2(1) = 5.36, **p = 0.02** |
|  | 2000 | 0.39 | 0.11 | χ2(1) = 11.80, **p < 0.001** |
|  | 2010 | 0.29 | 0.1 | χ2(1) = 10.99, **p < 0.001** |
|  | 2019 | 0.33 | 0.11 | χ2(1) = 11.16, **p < 0.001** |
| Shoreline | 1990 | 0.66 | 0.16 | **p < 0.001*** |
|  | 2000 | 0.76 | 0.16 | χ2(1) = 25.52, **p < 0.001** |
|  | 2010 | 0.6 | 0.14 | χ2(1) = 21.13, **p < 0.001** |
|  | 2019 | 0.61 | 0.14 | χ2(1) = 22.05, **p < 0.001** |
| County (random) | 1990 | — | 0.24 | — |
|  | 2000 | — | < 0.001 | — |
|  | 2010 | — | 0.16 | — |
|  | 2019 | — | 0.068 | — |
| Tract (random) | 1990 | — | 0.11 | — |
|  | 2000 | — | < 0.001 | — |
|  | 2010 | — | < 0.001 | — |
|  | 2019 | — | < 0.001 | — |

**
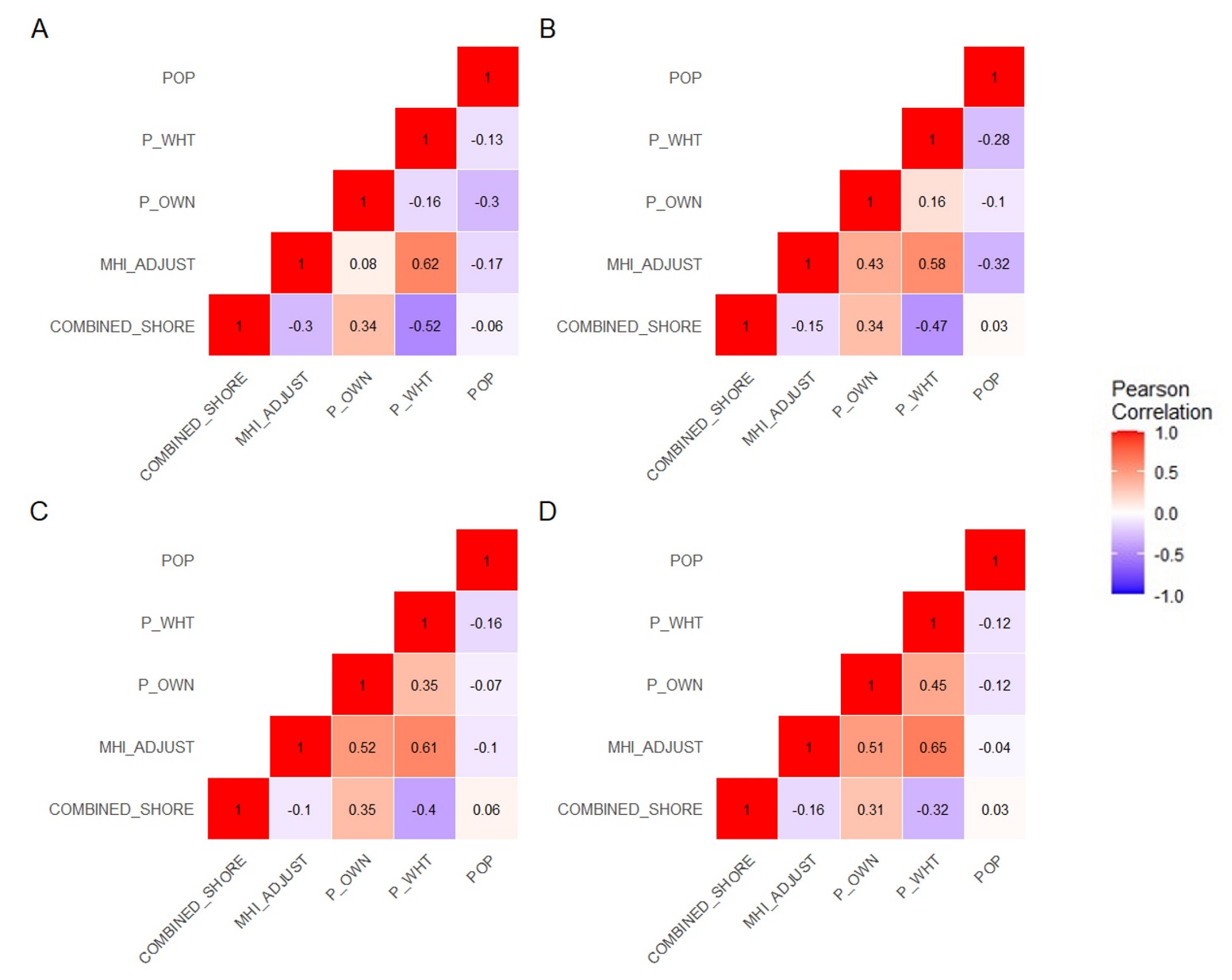
Fig. S7** Correlation heatmaps of predictor variables at the tract-level in 1990 (A), 2000 (B), 2010 (C), and 2019 (D). Numbers in cells represent values of Pearson correlation coefficients. Abbreviations: COMBINED_SHORE = shoreline length, MHI_ADJUST = median household income (adjusted to 2010 USD), P_OWN = % homeowners, P_WHT = % White, POP = population.
